# Addressing overlapping sample challenges in genome-wide association studies: Meta-reductive approach

**DOI:** 10.1371/journal.pone.0296207

**Published:** 2024-08-01

**Authors:** Farid Rajabli, Azra Emekci

**Affiliations:** 1 John P. Hussman Institute for Human Genomics, University of Miami Miller School of Medicine, Miami, FL, United States of America; 2 Dr. John T Macdonald Foundation Department of Human Genetics, University of Miami Miller School of Medicine, Miami, FL, United States of America; 3 Pioneer High School, San Jose, CA, United States of America; Universidad de la Republica Uruguay: Facultad de Ingeniería, URUGUAY

## Abstract

Polygenic risk scores (PRS) are instrumental in genetics, offering insights into an individual level genetic risk to a range of diseases based on accumulated genetic variations. These scores rely on Genome-Wide Association Studies (GWAS). However, precision in PRS is often challenged by the requirement of extensive sample sizes and the potential for overlapping datasets that can inflate PRS calculations. In this study, we present a novel methodology, Meta-Reductive Approach (MRA), that was derived algebraically to adjust GWAS results, aiming to neutralize the influence of select cohorts. Our approach recalibrates summary statistics using algebraic derivations. Validating our technique with datasets from Alzheimer disease studies, we showed that the summary statistics of the MRA and those derived from individual-level data yielded the exact same values. This innovative method offers a promising avenue for enhancing the accuracy of PRS, especially when derived from meta-analyzed GWAS data.

## Introduction

Polygenic risk scores (PRS) have emerged as an essential tool in the field of genetics [1, 2]. These scores offer a unique insight into an individual’s genetic predisposition to a wide array of diseases and traits, capturing the cumulative effects of multiple genetic variants [3]. The Genome-Wide Association Studies (GWAS) serve as the base for creating PRS [4]. GWAS investigates the entire genetic makeup of individuals to identify genetic variations associated with specific diseases or traits. The predictive accuracy and precision of PRS are enhanced when the base GWAS summary statistics come from a sizeable sample, and the population in the GWAS matches the population where the PRS is being applied [4, 5]. Due to this need for a substantial sample size, studies often aim to meta-analyze all available genetic datasets to achieve the statistical power necessary for identifying genetic markers linked to the trait or disease. However, this approach presents a challenge in securing independent datasets for training, testing, and validating PRS performance [6]. The use of overlapping samples can inflate the PRS calculations, resulting in imprecise risk predictions.

A logical approach might be to exclude a specific cohort of interest and then rerun meta-analyses with the remaining datasets. However, given the significant computational resources needed and the difficulties in accessing detailed summary statistics for all cohorts, this isn’t always viable. Nonetheless, we do have access to the cohort-level data for the specific dataset we aim to employ as a training and testing set. Recognizing this advantage, we formulated an alternative technique that incorporates the cohort-level result of our chosen dataset along with the meta-analysis GWAS findings. The goal is to neutralize the impact of the overlapping cohort of interest on the meta-analysis GWAS summary statistics, thus producing a PRS that avoids the inflationary tendencies arising from overlapping samples.

In this study, we derived equations to adjust GWAS results, effectively eliminating the impact of selected cohorts in inverse variance-based fixed effect meta-analysis (FEMA) studies. Through comprehensive simulations and real data analysis, we demonstrated that our methodology effectively updates the base data’s summary statistics, thereby addressing the challenge.

## Materials and methods

### Derivation of adjusted summary statistics: Meta-Reductive Approach (MRA)

We analyzed two distinct sets of summary statistics:

1. A compilation from *n* datasets meta-analyzed using an inverse variance-based approach [7].
2. A specific dataset of interest that was also part of the meta-analysis.

For these datasets:

- B and *SE* symbolize the effect size and standard error, respectively, from the aggregate meta-analysis across *n* datasets.
- *β*_*i*_ and *se*_*i*_ specify the effect size and standard error for the individual cohort *i*.

Our primary aim was to compute a summary statistic that eliminates the influence of the dataset of interest, providing a clearer perspective on the overarching genetic structure.

- **Inverse-variance-weighted effect-size estimation**. The inverse variance method gives more weight to studies with smaller variance because they offer more precise estimates. The weight, *w*_*i*_, is the inverse of the variance, or squared standard error, of the effect size, *β*_*i*_.
  Given,
  $B=\frac{\sum_{i}^{n}\beta_{i}w_{i}}{\sum_{i}^{n}w_{i}}$ where the $w_{i}=\frac{1}{{{se}_{i}}^{2}}$
  Expanding this:
$$Bw_{1}+Bw_{2}+Bw_{3}+\cdots +Bw_{n-1}+Bw_{n}=\beta_{1}w_{1}+\beta_{2}w_{2}+\beta_{3}w_{3}+\cdots +\beta_{n-1}w_{n-1}+\beta_{n}w_{n}$$
  This is the weighted sum of the effect sizes across all datasets, including the one of interest.
  Now, to remove the effect of the specific dataset, *β*_*n*_, we rearrange:
$${Bw_{1}+Bw_{2}+Bw_{3}+\cdots +Bw_{n-1}+Bw_{n}-\beta_{n}w_{n}=\beta }_{1}w_{1}+\beta_{2}w_{2}+\beta_{3}w_{3}+\cdots +\beta_{n-1}w_{n-1}$$
  Which yields:
$$B+\frac{Bw_{n}-\beta_{n}w_{n}}{w_{1}+w_{2}+w_{3}+\cdots +w_{n-1}}=\frac{\beta_{1}w_{1}+\beta_{2}w_{2}+\beta_{3}w_{3}+\cdots +\beta_{n-1}w_{n-1}}{w_{1}+w_{2}+w_{3}+\cdots +w_{n-1}}$$
  This equation essentially adjusts the overall effect size, *B*, by subtracting the influence of the dataset of interest.
- **Standard error derivation**. The standard error (SE) offers a measure of the statistical accuracy of an estimate. Here, we adjust the SE based on the weights of all datasets excluding the one of interest.
  Using:
$${SE}^{2}=\frac{1}{w_{1}+w_{2}+w_{3}+\cdots +w_{n-1}+w_{n}}$$
  We derive:
$$w_{1}+w_{2}+w_{3}+\cdots +w_{n-1}=\frac{1-{SE}^{2}w_{n}}{{SE}^{2}}$$
  This equation gives the combined weight of all datasets, excluding the dataset of interest.
- **Adjusted effect size and standard error**. Post removing the influence of the dataset of interest, the modified effect size is given by:
$$B_{adj}=\frac{\beta_{1}w_{1}+\beta_{2}w_{2}+\beta_{3}w_{3}+\cdots +\beta_{n-1}w_{n-1}}{w_{1}+w_{2}+w_{3}+\cdots +w_{n-1}}=B+\frac{{SE}^{2}(Bw_{n}-\beta_{n}w_{n})}{{1-SE}^{2}w_{n}}$$
  This adjusted beta, *B*_*adj*_, having nullified the contribution of the specific dataset *n*.
  Additionally, the adjusted standard error is:
$${SE}_{adj}^{2}=\frac{{SE}^{2}}{1-{SE}^{2}w_{n}}$$
  This adjustment ensures that the standard error reflects the precision of our new effect size estimate, free from the influence of the specific dataset.

Ethical approval was not required for this study as it utilized publicly available summary statistics.

## Results

### Validation using real data

To validate our methodological approach, we utilized summary statistics from four publicly accessible Alzheimer disease studies: Kunkle et al. [8], Kunkle et al. [9] AA, Bellinguez et al. [10], and Moreno-Grau S. et al. [11] From these studies, 100,000 markers were selected to conduct a meta-analysis using the METASOFT software [12].

Following the initial meta-analysis, we applied a systematic "leave-one-out" strategy. For each iteration, we excluded the summary statistics from one dataset and conducted a meta-analysis of the remaining three. The results from this procedure served as our individual-level data for the three datasets in question.

**Fig 1:**
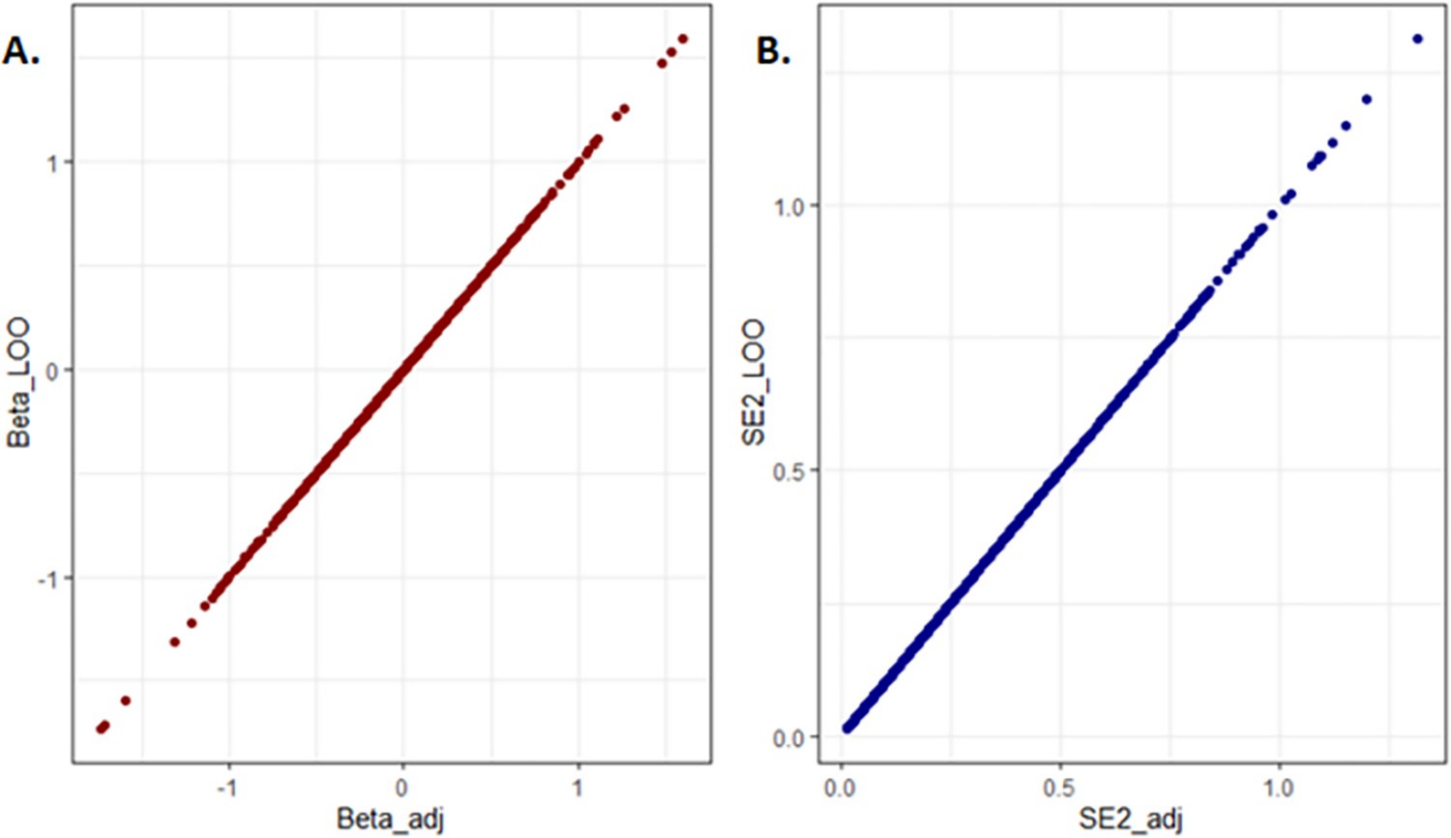
Comparison between the adjusted results from the Meta-Reductive Analysis (MRA) (Beta_adj and SE2_adj) approach and the "leave-one-out" inverse variance-based fixed effect meta-analysis (FEMA) (Beta_LOO and SE2_LOO). The MRA-adjusted values show identical results with the FEMA calculation for both Beta values (A) and Standard Error (B).

For the final step of validation, we calculated the adjusted *B*_*adj*_ and ${SE}_{adj}^{2}$ values based on MRA and compared them against the individual-level data summary statistics derived from the "leave-one-out" FEMA. Our results showed that the summary statistics of the FEMA and MRA approaches yielded the exact same values. To demonstrate this, we plotted the betas and standard errors (Fig 1). The graphical representation illustrates that both beta and standard error values from the “leave-one-out”“FEMA and MRA give the same results.

Additionally, we conducted a validation analysis for the APOE4 allele, utilizing data from a multi-ancestry study by Rajabli et al. [13], which included four population-based studies: African American, East Asian, Hispanic, and non-Hispanic Whites (S1 Table). We applied a "leave-one-out" strategy by removing one population at a time and performing the validation on the remaining studies. We followed the same steps as described previously, and the results were exact same across all tests, as detailed in Table 1.

**Table 1:** Validation analysis for APOE4 allele using summary statistics from Rajabli et al. study. "MRA-Beta" and "MRA-SE" denote beta and standard error values derived using the MRA approach, respectively. "Traditional-Beta" and "Traditional-SE" refer to beta and standard error values obtained from the meta-analysis of three studies.

Study removed	MRA-beta	MRA-SE	Traditional-Beta	Traditional-SE
**African American**	1.189192599	0.01671915	1.189192599	0.01671915
**East Asian**	1.09385844	0.017205134	1.09385844	0.017205134
**Hispanic**	1.215580078	0.016903311	1.215580078	0.016903311
**non-Hispanic White**	1.177899334	0.026321213	1.177899334	0.026321213

### Simulation

We simulated Beta coefficients and their corresponding SEs across ten studies, each containing 10,000 markers (using R programming language.) We generated random Beta coefficients utilizing the “**rnorm”** function, under the assumption of a normal distribution, characterized by a mean of zero and a standard deviation of one. We used “**runif”** function to produce random SEs values from a uniform distribution, with specified minimum and maximum limits of 0.1 and 0.5, respectively. The simulated Betas and SEs for each marker within a study were then organized into dedicated columns within a data frame.

We applied the “**rma”** function from the **metaphor [14]** package to facilitate a fixed-effects meta-analysis on the generated Betas and SEs. Then we implemented "leave-one-out" strategy, mirroring the methodology applied to real data. We calculated the adjusted *B*_*adj*_ and ${SE}_{adj}^{2}$ values employing our proposed method and compared it with individual-level data derived from the "leave-one-out" meta-analyses. The outcomes revealed that the summary statistics were identical, similar to the findings from real data analysis. The simulation script is provided with the MRA function here: https://github.com/hihg-um/MRA.

## Discussion

This study employs algebraic adjustments to GWAS summary statistics to eliminate the influence of specific datasets in meta-analyses. The algebraic solutions applied to real and simulated data consistently matched our expectations of achieving identical results. The validation confirms the robustness and reliability of derived equations, emphasizing the effectiveness of our methods in addressing the challenges associated with sample overlap in meta-analyses.

Furthermore, our approach utilizes the widely recognized inverse-variance method for fixed-effect meta-analysis. This choice ensures that our adjustments are based on a widely accepted framework, enhancing the general applicability and relevance of our findings. While our study focuses on inverse-variance method fixed-effects models, the foundational principles of our approach could potentially be adapted for random-effects meta-analyses, which would be useful in situations where variability between studies is significant.

In summary, our research highlights the practicality of excluding specific datasets to refine effect estimates in inverse-variance method meta-analysis. We provide a method that enables researchers to neutralize the impact of overlapping cohorts on meta-analysis GWAS summary statistics, thereby producing a PRS that avoids the possible inflations associated with overlapping samples. This approach is important for enhancing the accuracy and reliability of PRS in genetic studies.

## Supporting information

S1 Table Summary statistics for APOE e4 allele association with Alzheimer disease across populations. (XLSX)
